# Microbiome modulation in inflammatory diseases: Progress to microbiome genetic engineering

**DOI:** 10.1186/s12935-023-03095-2

**Published:** 2023-11-11

**Authors:** Fatemehsadat Mousavinasab, Ronika karimi, Sima Taheri, Fatemeh Ahmadvand, Saameh Sanaaee, Sajad Najafi, Masood Soltani Halvaii, Alireza Haghgoo, Marzieh Zamany, Jamal Majidpoor, Mina Khosravifar, Mohammad Baniasadi, Mehrdad Talebi, Abolfazl Movafagh, Seyed Mohsen Aghaei-Zarch, Nastaran Khorram, Poopak Farnia, Kambiz Kalhor

**Affiliations:** 1 Institute for Biomedical Sciences, Georgia State University, Atlanta, GA USA; 2 Department of Cellular and Molecular Biology, Faculty of Advanced Science and Technology, Tehran Medical Sciences, Islamic Azad University, Tehran, Iran; 3 Department of Microbiology, Shahr Qods Branch, Islamic Azad University, Tehran, Iran; 4 Department of Biology, Islamic Azad University, Tehran, Iran; 5 Department of New Science, Faculty of Cellular and Molecular biology, Tehran Medical Branch, Islamic Azad University, Tehran, Iran; 6 Department of Medical Biotechnology, School of Advanced Technologies in Medicine, Shahid Beheshti University of Medical Sciences, Tehran, Iran; 7 Faculty of Biological Sciences, Kharazmi University, Tehran, Iran; 8 Department of Microbiology and Microbial Biotechnology, Faculty of Life Sciences and Biotechnology, Shahid Beheshti University, Tehran, Iran; 9 Shahid Akbarabadi Clinical Research Development Unit, Iran University of medical Science, Tehran, Iran; 10 Department of Anatomy, Faculty of Medicine, Infectious Disease Research Center, Gonabad University of Medical Sciences, Gonabad, Iran; 11 Institute of Diabetes, Obesity, and Metabolism, Perelman School of Medicine, University of Pennsylvania, Philadelphia, PA 19104 USA; 12 Department of Basic Sciences, School of Medicine, Bam University of Medical Sciences, Bam, Iran; 13 Department of Medical Genetics, School of Medicine, Shahid Sadoughi University of Medical Sciences, Yazd, Iran; 14 Department of Medical Genetics, School of Medicine, Shahid Beheshti University of Medical Sciences, Tehran, Iran; 15 Department of Chemical and Petroleum Engineering, Sharif University of Technology, Tehran, Iran; 16 Mycobacteriology Research Center, National Research Institute of Tuberculosis and Lung Disease, Shahid Beheshti University of Medical Sciences, Tehran, Iran; 17 Department of Earth and Planetary Sciences, University of Tennessee, Knoxville, USA

**Keywords:** Microbiome, Genetic Engineering, Inflammatory diseases

## Abstract

Recent developments in sequencing technology and analytical approaches have allowed researchers to show that the healthy gut microbiome is very varied and capable of performing a wide range of tasks. The importance of gut microbiota in controlling immunological, neurological, and endocrine function is becoming well-recognized. Thereby, numerous inflammatory diseases, including those that impact the gastrointestinal system, as well as less obvious ones, including Rheumatoid arthritis (RA), cancer, gestational diabetes (GD), type 1 diabetes (T1D), and type 2 diabetes (T2D), have been linked to dysbiotic gut microbiota. Microbiome engineering is a rapidly evolving frontier for solutions to improve human health. Microbiome engineering seeks to improve the function of an ecosystem by manipulating the composition of microbes. Thereby, generating potential therapies against metabolic, inflammatory, and immunological diseases will be possible through microbiome engineering. This essay first provides an overview of the traditional technological instruments that might be used for microbiome engineering, such as Fecal Microbiota Transplantation (FMT), prebiotics, and probiotics. Moreover, we will also discuss experimental genetic methods such as Metagenomic Alteration of Gut microbiome by In situ Conjugation (MAGIC), Bacteriophage, and Conjugative plasmids in manipulating intestinal microbiota.

## Introduction

The human microbiome is full of mysteries and plays a significant role in the general well-being of the host’s immunity, metabolism, and digestion [1]. Generally, approximately 500–1,000 species of viruses, bacteria, protozoa, and fungi inhabit the human body. Every individual has a particular microbiome composition, and these differences are much more significant compared to the usual biochemical differences within a person [2].

**Fig. 1:**
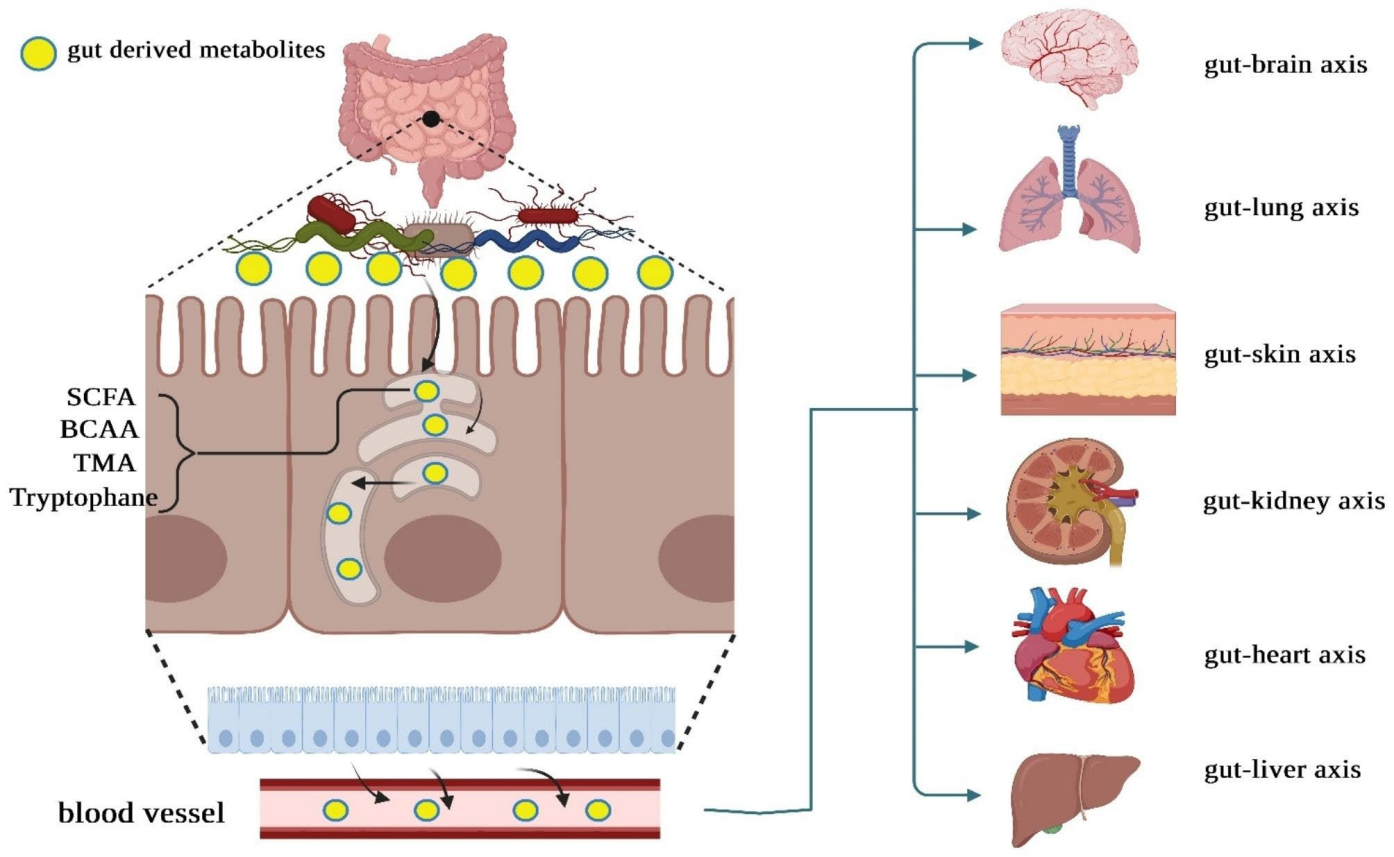
The continuous movement of gut bacteria-derived metabolites, including short-chain fatty acids (SCFA), branched-chain amino acids (BCAA), trimethylamine N-oxide (TMA), tryptophan in human hosts from the intestinal mucosa to other extraintestinal sites. Gut microbiota-derived metabolites have a central role in the physiology and homeostasis of the body. They are implicated in the pathogenesis of complex inflammatory diseases such as diabetes and cancer

The gut microbiota carry out various functions influencing the host’s overall health, including nutrient metabolism and immune system regulation [3]. Various bioactive compounds are synthesized by human gut microbiota powered by dietary nutrients. It is also possible for microbial metabolites to signal other organs within the host body, enabling them to communicate with hormones, the immune system, metabolic processes, and other functions within the host [4]. In this regard, Ma et al. conducted an experimental study to evaluate the effectiveness of sodium butyrate (NaB) - a prominent byproduct of microbial fermentation in the gut - in regulating the gut microbiota of mice with colorectal cancer (CRC) liver metastasis (CLM). In an intrasplenic tumor injection model of BALB/c mice, the addition of sodium butyrate (NaB) supplements reduced liver metastasis in CT26 colon cancer cells. Through the 16 S rRNA gene sequencing approach, a modified microbiota composition has been detected in CLM mice. Elevated levels of *Firmicutes* and *Proteobacteria* define this alteration. The dysbiosis observed in CLM mice was positively impacted by the administration of NaB. Upon conducting a functional analysis of the KEGG pathways, it was observed that NaB effectively modified pathways associated with immune system diseases and primary immunodeficiency in the CLM mice. In addition, in the liver of CLM mice, NaB was found to reduce the levels of T regulatory cells while simultaneously increasing the levels of natural killer T cells and T helper 17 cells. Consequently, there was a decrease in the secretion of IL-10 and an increase in the secretion of IL-17. Therefore, the administration of NaB in CLM mice resulted in a positive modulation of gut microbiota and an improvement in the host immune response [5]. In addition to nutrient metabolism, a substantial body of research, encompassing studies conducted in both animal and human subjects, has consistently demonstrated the pivotal relationship between the gut microbiota and inflammatory processes [6]. The gut microorganisms can enzymatically break down complex carbohydrates into SCFAs. SCFAs are pivotal in the intricate interplay among dietary components, the gut microbiota, and subsequent inflammatory pathways. *Faecalibacterium prausnitzii* has been recognized as a bacterium capable of producing butyrate and exhibits an inverse correlation with various pro-inflammatory markers. Butyrate is an anti-inflammatory metabolite recognized for suppressing the pathways generating pro-inflammatory cytokines. Additionally, butyrate reduces the likelihood of insulin resistance development by enhancing insulin signaling [7]. Furthermore, studies have demonstrated that butyrate can mitigate the translocation of lipopolysaccharides (LPS) within the intestinal tract, thereby diminishing the consequential effects associated with LPS. Thereby, perturbation to the gut microbiome has the potential to give rise to prolonged or persistent inflammation, which could consequently contribute to the onset of inflammatory conditions, including inflammatory bowel disease, diabetes, or cancer (Fig. 1) [8].

The Human Microbiome Project (HMP) and the advancement of omics technologies have substantially contributed to elucidating microbial composition and acquiring insights into microbiomes [9]. Microbiome engineering is primarily implemented in the context of the human microbiome, as it holds significant promise for the therapeutic management of diseases. This is attributable to the capacity to modulate human microbiota, which has been shown to influence the host’s physiological processes and its compositional links to various diseases and disorders, including diabetes and cancer [10, 11]. As a result, there is an increasing enthusiasm for microbiome engineering to influence the composition of microbiota in order to enhance host functionality and promote human well-being. Here, we present a comprehensive survey of the existing technological tools that can be employed in microbiome engineering. These tools also offer the intriguing potential to delve into the development of targeted interventions and personalized therapeutic approaches for diseases associated with the microbiome, such as diabetes and cancer.

### Microbiome composition and its variation

Gut microbiota comprises different bacteria species taxonomically classified by genus, family, order, and phyla [12, 13]. Only a few phyla are represented, accounting for more than 160 species from a taxonomical perspective classified by genus, family, order, and phyla. The central gut microbial phyla are *Firmicutes*, *Bacteroidetes*, *Actinobacteria*, *Proteobacteria*, *Fusobacteria*, and *Verrucomicrobia*, and the two phyla *Firmicutes* and *Bacteroidetes* being the dominant ones representing up to 90% of gut microbiota. The *Firmicutes* phylum exceeds 200 genera, such as *Lactobacillus*, *Bacillus*, *Clostridium*, *Enterococcus*, and *Ruminococcus*. A significant part of *Firmicutes* phyla is formed by *Clostridium* (95%). *Bacteroidetes* are composed of two dominant genera such as *Bacteroides* and *Prevotella*. The *Actinobacteria* phylum includes a minor proportion of the whole and is commonly represented by the *Bifidobacterium* genus [14]. Importantly, each anatomical section of the intestine has its physiological characteristics. In this context, the large intestine, with its low metabolic rate and neutral to slightly acidic pH, is home to the largest microbial population in the body, which is dominated by obligate anaerobic bacteria. Meanwhile, due to its relatively short transit times (3–5 h) and high concentrations of bile, the small intestine presents a more challenging environment for microbes to colonize. So, the small intestine harbors a microbial community with less diversity and abundance. Multiple factors that ultimately influence gut microbiome compounds have been identified, such as seasonal variations in both (i) the abundance of specific taxa containing extremely abundant phyla *Bacteroidetes* and *Firmicutes*, also (ii) total gut microbiome diversity [15]. One explanation for microbiome composition differences is a dietary fluctuation between seasons. These findings demonstrated the plastic nature of the human gut microbiome in response to variations in diet [16]. Besides, a large cross-sectional study by O Manor et al. provided a deeper analysis of the relationship between host-driving factors and the gut microbiome. This study was done on about ~ 3400 healthy US individuals. Diet, medication use, and clinical blood markers were identified as lifestyle and clinical factors influencing the gut microbiome’s composition, including individual taxonomies, diversity, and inferred functional pathways. Major axes of taxonomic variance in the gut were revealed, and the maximum diversity was discovered along the *Firmicutes*-to-*Bacteroidetes* axis. They also revealed that the host itself develops its microbiome community, which is robust and completely independent from the diverse subcategory species of microbial associations [17]. In another study by KVA Johanson et al., diversity analysis of gut microbiome disclosed that individuals with more personal relationships and interactions are prone to have a more diverse microbiome. This suggests that social interactions may affect determining the human gut microbiome composition. Their further analysis also disclosed that higher stress and anxiety levels are directly connected to lower diversity and can change the microbiome community [18]. Another critical factor in determining diversity in microbiome composition has been claimed to be the host’s genetic profile. Therefore, the genetic compound of each individual can be the real explanation behind these differences. However, the actual mechanism behind these links still needs to be clarified. The crucial role of metabolic functions and the immune system was demonstrated by surveying probable instruments involved in the host’s genetic profile shaping microbiome composition [19].

### Microbiome and inflammation

The host immune system has been demonstrated to significantly impact gut microbiota development and function. Conversely, host-associated microorganisms contribute considerably to the development and function of innate and adaptive immunity by establishing a “tolerant” phenotype that facilitates the continuation of host-microbe co-existence [20]. Thereby, there is a bidirectional crosstalk between the immune response and the host’s commensal microorganisms. In this context, recent investigations explore the interplay among the local immune responses and intestinal microbiota. It was observed that there is a significant positive correlation between IL-18 and the two genera *Mycoplasma* and *Mesoplasma*, both members of the class Mollicutes [phylum Tenericutes]. It was validated that Mycoplasma induces the expression of pro-inflammatory cytokines in monocytic cells and that lipid membrane constituents are largely responsible for these effects. We further observed a relatively weak anti-correlation between members of the class *Flavobacteria* and both IL-8 and IL-4. A possible candidate for this trend is the genus *Carnobacterium* [a lactic acid bacterium producing bacteriocin-like compounds] [21]. The direct communication of microbiota with the host has been exhibited to occur through highly conserved structural components, microbe-associated molecular patterns (MAMPs), which are recognized by the host and include lipopolysaccharides (LPSs), peptidoglycan (PGN), and flagellin. Immune cells and intestinal epithelial cells (IECs) produce pattern-recognition receptors (PRRs) that bind to MAMPs as a primary method of their recognition by the host. Nucleotide-binding oligomerization domain (NOD)-like receptors (NLRs) and Toll-like receptors (TLRs) are members of the PRRs, a diverse group of cytoplasmic and transmembrane nonspecific immune receptors [22]. Intracellular signaling cascades could be triggered through the stimulation of PRRs, which could induce immunomodulatory molecule expression, thereby arranging early immune responses and mucosal inflammation and further triggering nonspecific and specific immune pathways. While pathogens and pathobionts activate PRR to initiate pro-inflammatory signaling cascades, commensal microbiota can also use similar mechanisms to dampen inflammation and promote intestinal homeostasis [23]. Notably, the host immune system is perpetually influenced by the gut microbiota to maintain the intestinal homeostasis and symbiosis of the host and microbiome. MAMPs and microbiota-derived compounds, for example, can alter host immune responses and modulate mucosal barrier function by activating NLR complexes termed inflammasomes. The microbiota activates signaling of NOD-, leucine-rich repeat (LRR)-, and pyrin domain containing 6 (NLrP6) inflammasomes to induce the steady-state secretion of pro-inflammatory IL-18 and, consecutively, activating AMP and production of mucin in intestinal mucosa that could lead to refinement of microbiota composition [24]. In addition, SCFAs activate NLRP3 through GPR43 and GPR109A, resulting in mucosal IL-18 production. Notably, the gastrointestinal microbiota has recently been identified as a significant contributor to the regulation of immune effector cell maturation and activity, and dysbiosis has been shown to contribute to intestinal mucosa permeability and the induction of innate defenses, making it a candidate environmental risk factor capable of triggering a variety of inflammatory diseases such as diabetes and cancer [25].

### Microbiome and inflammatory diseases

**Fig. 2:**
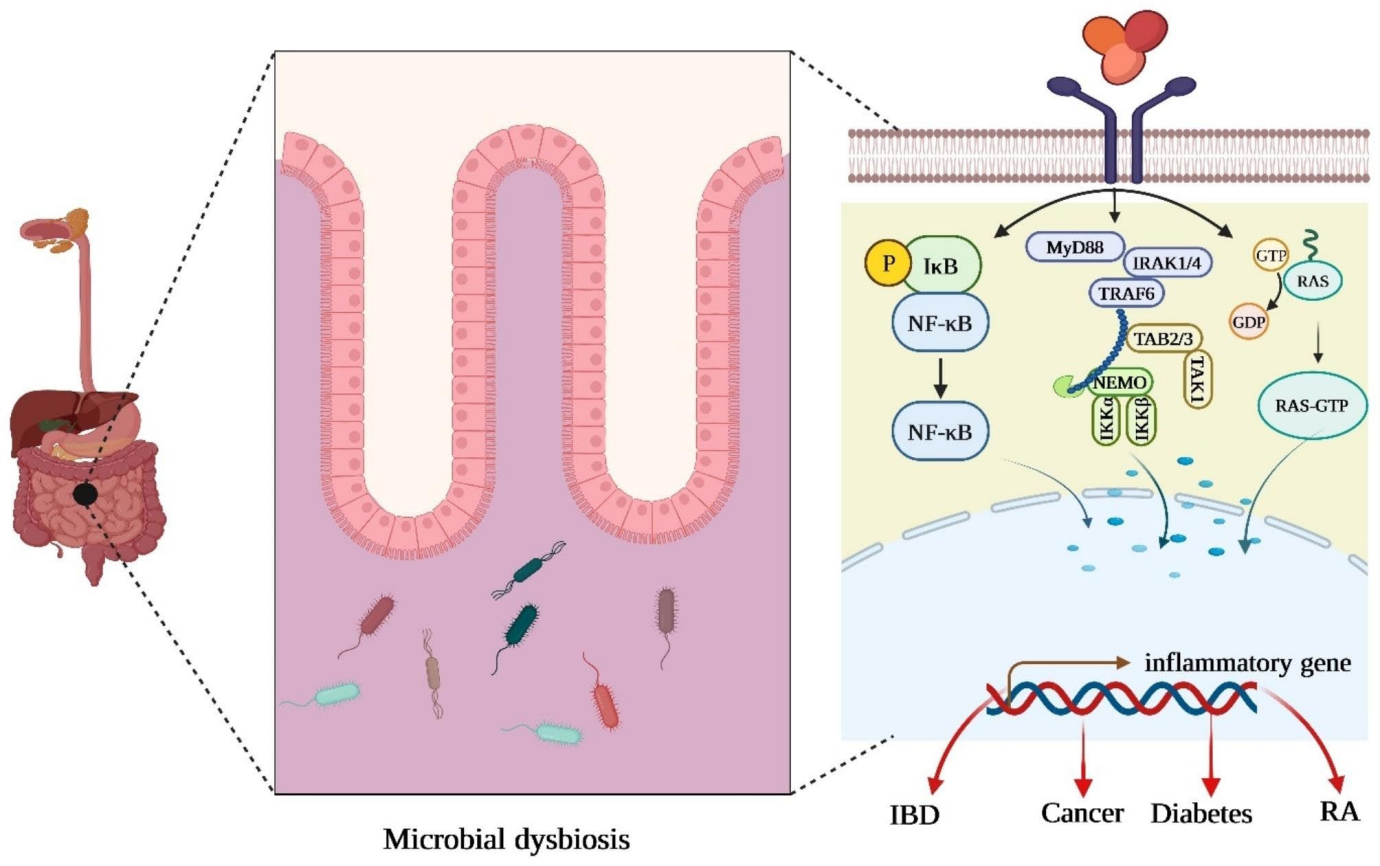
Schematic representation of microbiome dysregulation in inflammatory diseases. The host immune system plays an essential role in shaping the gut microbiota, and reciprocally, host-associated micro-organisms significantly influence the development and function of innate and adaptive immunity. It was found that a disrupted microbial community leads to the activation of an inflammatory response and is linked to various pathological conditions such as inflammatory bowel disease, rheumatoid arthritis, diabetes, and even cancer [52–54]

The causes of inflammatory diseases are multifactorial and include age, genetics, and environment. Microorganisms are crucial in maintaining gastrointestinal homeostasis and can potently modulate systemic immunity, and differences in the microbiota have been observed in patients with inflammatory diseases compared to healthy controls. A growing amount of clinical research is being done better to understand the microbial community’s role in inflammatory diseases (Fig. 2) [26, 27].

Diabetes mellitus (DM) is a diagnostic term for chronic metabolic diseases characterized by abnormal glucose homeostasis resulting in elevated blood sugar [28–30]. In the past ten years, the importance of gut microbiome in DM pathogenesis has attracted much attention worldwide [31–36]. In this regard, Das et *al.* investigated whether the abundance of pro-inflammatory and anti-inflammatory bacteria in the gut microbiomes of people with DM and DR changed. They observed decreased anti-inflammatory bacteria (*Roseburia*, *Lachnospira*, *Coprococcus*, *Phascolarctobacterium*, *Blautia*, and *Anaerostipes*). In contrast, the pro-inflammatory bacteria (*Escherichia*, *Enterobacter*, *Methanobrevibacter*, and *Treponema*) were more abundant in T2DM than healthy controls (HC). They also observed an increase of a few pro-inflammatory and anti-inflammatory bacteria in HC and T2DM, respectively. Their findings show that a balance of anti- and pro-inflammatory bacteria is critical for HC, but there must be a predominance of anti-inflammatory bacteria over pro-inflammatory bacteria. Furthermore, in their study, T2DM patients had lower levels of anti-inflammatory gut microbiota (*Roseburia*, *Lachnospira*, *Coprococcus*, *Phascolarctobacterium*, *Blautia*, and *Anaerostipes*). In addition to the genera such as *Roseburia*, *Lachnospira*, and *Blautia*, numerous additional anti-inflammatory genera like *Faecalibacterium*, *Bifidobacterium*, *Ruminococcus*, *Mitsuokella*, *Streptococcus*, *lactobacillus*, and *Butyrivibrio* were also reduced in DR. In addition, they found that the pro-inflammatory bacterium *Sutterella*, as well as various potentially harmful bacteria (*Clostridium*, *Haemophilus*, *Erwinia*, *Desulfovibrio*, *Bulleida*, *Rothia*, and *Comamonas*) and probiotic bacterium *Lactobacillus*, were lower in DR patients compared to HC and T2DM. Thus, gut microbiota dysbiosis that promotes inflammation is a general feature of T2D and diabetes-related comorbidities [37].

Cancer is considered the leading cause of death worldwide, prompting extensive scientific inquiry into molecular mechanisms, treatment modalities, and associated prognostic factors [38–43]. Recent experimental investigations confirm a direct relationship between the microbiome and cancer [44–47]. Carcinogenesis can be enhanced by microbiota dysbiosis in three different ways. Firstly, secreted genotoxins can be harmful to the host’s DNA. Secondly, inflammation happens due to the toxins and metabolites produced by microbiota. Thirdly, the abnormally regulated immune system responding to various microbes could initiate cancer [48]. In a survey by RM Ferreira et al., the compounds of the gastric microbiota in chronic gastritis and gastric carcinoma were evaluated. Their findings showed decreased microbial diversity and a considerable drop in the density of Helicobacter. In contrast, they witnessed the enrichment of other genera, demonstrated mainly by intestinal commensals in gastric carcinoma microbiota. Additionally, they concluded that dysbiosis could discriminate between gastritis and gastric carcinoma [49]. In research by J Zhang et al., thyroid endocrine disorders such as thyroid cancer and thyroid nodules association with the gut microbiome were investigated. This study identified higher abundances of *Neisseria* and *Streptococcus* for thyroid cancer and thyroid nodules via 16 S rRNA (16 S ribosomal RNA) gene-based sequencing protocol. However, *Butyricimonas* and *Lactobacillus* demonstrated particularly less relative abundance for thyroid cancer and thyroid nodules. Thus, thyroid cancer and thyroid nodules are closely related to the gut microbiome composition [50]. In addition, Niccolai et al. explored the immunity–microbiota axis in human CRC, comparing the distribution of the cytokine profile and the GM composition in cancerous and surrounding mucosa. Their data describe an apparent dissimilarity of the cellular and molecular inflammatory profile and intestinal microbiota composition between the tumor and the adjacent healthy tissue, displaying the generation of a peculiar CRC microenvironment. Their further experimentation disclosed that microbial communities can drive and modulate the antitumor immune response. They finally observed that *Prevotella* and *Bacteroides* species are correlated positively and negatively, respectively, with the IL-9 that has an intriguing and still debated role in tumor immunity. In this manner, their findings confirm the presence of bidirectional crosstalk between the immune response and the host’s commensal microorganisms [51].

### Microbiome engineering

The application of microbiome knowledge is mainly focused on treating and preventing dysbiosis and related disorders. However, due to a lack of complete understanding, it has not been used to its full potential, and a significant portion of the human microbiota has yet to be discovered [55]. As a result, identifying dominant or unique members of microbial communities performing specific therapeutic roles is expected to bridge this gap and become the backbone of bacteriotherapy for various illnesses in the future. The aim of microbiome engineering is the refinement of an ecosystem’s function through alterations in microbial composition. Microbiome engineering can also alter microbial compositions to improve host characteristics. So, the possibility of modifying gut bacterial ecology has become a principal aim, and various steps have been tried to identify appropriate populations. The success of microbiome engineering faces two fundamental hurdles: [1] the design of a microbiome with improved function and [2] the development of an improved microbiome in a recipient system of interest [56]. The direct methods of modifying the gut microbiome include using Fecal Microbiota Transplantation (FMT), prebiotics, and probiotics [57]. To change gut microbiota by FMT, a healthy donor’s microbiome was examined as a possible treatment targeted to restore healthy microbiota in the recipient. This is a microbiota-agnostic approach since the complete microbial species’ associations are transplanted from a healthy donor. Most importantly, the donor’s health must be examined before the transplant [58]. This method carries a high risk of transplanting unwanted microbes, which may cause more damage. Prebiotics are considered as another means besides FMT in microbiome engineering. A Prebiotic is a substrate elected by host microorganisms to bestow health benefits. Commonly known as prebiotics, they are utilized as modifiers of the gut microbiome that are non-digestible carbohydrates (NDC), including fructooligosaccharides, galactooligosaccharides, inulin, and oligosaccharides [59]. In studies involving *Bifidobacterium* containing prebiotics, oligosaccharides can produce fermentative end products, such as acetate and lactate. These products lead to better gut barrier integrity along with pathogen inhibition. Nowadays, the aim is to enhance the growth of available bacterial communities by using natural prebiotics and adjusting metabolic pathways present in the gut. However, the precise mechanisms of both methods are still unclear [60]. In addition to prebiotics, probiotics are also utilized as targeted modifiers in regulating the gut microbiome. Unlike prebiotics, these are living microorganisms and can benefit the host when applied in sufficient portions. Probiotics improve gut health through various mechanisms, including pH level modification and resistance to colonization. The gut microbiome is manipulated to alter its compositions by using probiotic supplements [61]. Although these established methods are effective for directing bioprocess engineering, they must allow for the precise manipulation and control of microbiomes required to realize their full potential. Over the last two decades, systems biology has changed our knowledge of the metabolic networks that drive microbiome activities, and more recently, genetic engineering methods for nonmodel microorganisms and microbiomes have begun to emerge [62].

We employ genetically modified bacteria as noninvasive tools in microbiome genetic engineering to assist available research and acquire insights into what could be happening in situ. “Chassis” is a term within synthetic biology, referring to the cell type keeping and maintaining DNA constructs for a particular purpose. The following factors influence the choice of chassis for interactions with the microbiome: [1] survivability, [2] colonization, [3] localization, and [4] genetic tractability. The viability of the chassis relates to whether it will survive through the gastrointestinal tract. The term “colonization” relates to whether the chassis will integrate with the natural gut bacteria, and certain chronic disorders require colonization for long-term therapy. Localization could be defined as the specific affected area of disease in the intestine. For instance, *Bacteroides* spp., which are localized in the cecum and colon, could also act as a means of ulcerative colitis treatment, only affecting the large intestine; in addition, *Lactobacillus* spp., localized in the small intestine, could be utilized for the treatment of Crohn’s disease, possibly affecting any region of the gastrointestinal tract. Finally, genetic tractability is the possibility of the organism being potently genetically modified through transformation, gene expression, or activation [60]. Despite this, the possibility of genetically engineering existing gut microbes to be compatible with the human immune system or the rest of the community has yet to be extensively investigated. Some gene editing tools have only been developed for individual species or bacterial composition. Such tools can upgrade the gut microbiome from association to causation and lay the grounds for future therapeutics [63].

### CRISPR as a powerful genetic tool in genetic engineering

Clustered Regularly Interspaced Short Palindromic Repeats (CRISPR), along with associated proteins, organize the adaptive immune system of bacteria, which makes them capable of protecting the bacteria against invasive nucleic acids [64]. The CRISPR-Cas system allows for incorporating foreign genetic material into the CRISPR array, which may be seen as a genomic index of repeated immunizations. CRISPR/Cas system is categorized generally into two different groups, which consist of five types (I, II, III, IV, V) with 16 subtypes (I-A, B, C, D, E, F, U; II-A, B, C; III-A, B, C, D; IV & V), predominantly laid on functions plus the architecture of specific Cas endonucleases with CRISPR. The most favored and broadly explored subtype is II-C CRISPR/Cas9. It is widely typical in most bacterial genomes and capable of sustaining omnipresent cas1 & cas2 like other subtypes along with cas9 protein-coding genes [65]. Nowadays, the CRISPR/Cas9 mechanism is considered a way of engineering probiotic strains for supplement therapies. In this regard, Zhou et al. exploited the heterologous Type II CRISPR-Cas9 system and the endogenous Type I-B CRISPR-Cas system in probiotic *C. butyricum* for seamless genome engineering. They realized that advancement in heterologous CRISPR-Cas9 and endogenous Type I‐B CRISPR‐Cas systems for genome editing in *C. butyricum* could drastically expand available gene engineering tools for this specific probiotic bacterium, which is also a valuable method for disclosing beneficial impacts of *C. butyricum* mechanisms, therefore encouraging discovering the full potential of multifunctional probiotic [66]. L Zheng et al. worked on developing a CRISPR/Cas-based genome editing tool that is versatile and very efficient, enabling gene deletion and insertion without any trace on the human gut microbiome. For this reason, multiple CRISPR/Cas systems were constructed in one Bacteroides–E. coli shuttle plasmid and the effectiveness of genome editing in *Bacteroides thetaiotaomicron* was evaluated, such as the mode of Cas protein expression (constitutive, inducible), various Cas proteins (FnCas12a, SpRY, SpCas9), and sgRNAs. They later proposed that CRISPR/FnCas12a can be widely used to engineer diverse gut Bacteroides species such as *Bacteroides fragilis*, *Bacteroides ovatus*, *Bacteroides uniformis*, and *Bacteroides vulgatus* [67]. Moreover, Shin et al. used CRISPR-Cas9 to implant the bacterium E. limosum, an essential bacteria from a biotechnological perspective. Through the CRISPR-Cas9 editing system, they showed the exact knock-out of the Wood-Ljungdahl (WL) pathway by homologous recombination. Subsequently, they examined the CRISPRi technique’s abilities to suppress some genes in the WL pathway and fructose-PTS system. These investigations indicate that advances in the CRISPR system and genetic tools can be multifunctional metabolic engineering tools for *E. limosum* [68]. In this regard, such tools supply innovative methods to study microbes thoroughly and clarify their parts in complex compositions, leading to innovative therapies such as microbiome engineering. There is a significant problem in the implementation of regulating microbiome members broadly, which is the complexity of introducing exogenous DNA in most bacterial species. Some bacteria are more or less amenable to electroporation, conjugation, or transduction techniques. However, most bacteria are facilitated by restriction systems that can perish incoming DNA. Another obstacle is related to the origin of replication, and if chosen incorrectly, bacteria may become incapable of replicating plasmid DNA. It is also worth mentioning that bacteria resistant to growing in a laboratory environment, including *Mycobacteria* and *Treponema*, are the most difficult to engineer [69].

### Major strategies used in microbiome genetic engineering

**Fig. 3:**
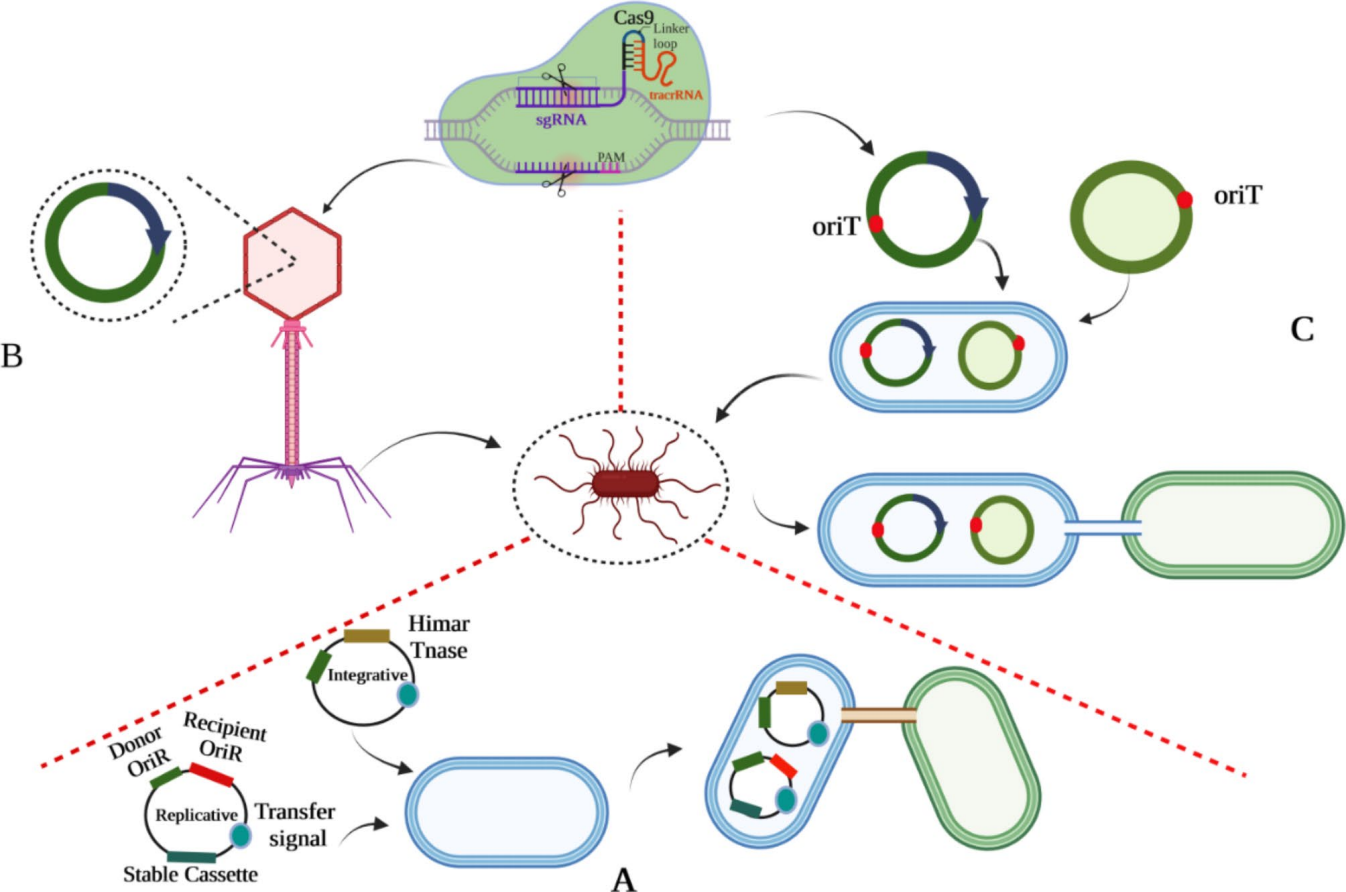
Major strategies in microbiome genetic engineering (A: MAGIC; B: Bacteriophage-mediated gene transfer; C: Conjugative plasmid gene transfer)

Genetic engineering begins with inducing DNA into the cell. The conventional transformation consists of electroporation, chemical transformation, and natural competence. Approximately 80 species of bacteria have been categorized as suitable for genetic engineering through in vitro methods. Each species requires special conditions for transformation, and creating such conditions for individual species demands a lot of effort [70]. In the following section, current genetic engineering approaches will be discussed (Fig. 3).

#### Conjugative plasmids

These circular antagonistic genomic elements regulate their transfer from one bacterium to another. Through proper oriT sites, these self-transmissible plasmids will be capable of co-transfer of non-conjugative plasmids. Non-conjugative plasmids can become mobile through conjugation when the relaxosome of the conjugative plasmid identifies the equivalent oriT site. Conjugative plasmids can operate independently without specific receptors on the host. They are relatively immune to bacterial defense systems (restriction-modification systems) because of the complementary strand with host-specific epigenetic modifications. Numerous factors, including concentrations of NaCl, butyrate, or propionate, epithelial cells, and inflammation, have been demonstrated to impact the transfer of certain conjugative plasmids in the intestinal microbiota. A methodical quantification of transfer rates was performed by Neil et al. in the intestinal tract of mice for 13 conjugative plasmids, which included ten significant incompatibility groups. Most of these plasmids exhibited limited or no ability to undergo conjugation under the investigated conditions or achieved only modest transfer rates. Surprisingly, the IncI2 conjugative plasmid TP114 was recognized as an effective vehicle for DNA transfer, displaying a high level of proficiency by successfully transferring to nearly all of the recipient bacteria examined. Furthermore, the data demonstrate that the I-complex conjugative plasmids’ type IV pilus is vital in TP114 transfer within the mouse intestinal microbiota. Its contribution is believed to be related to enhancing the stability of the mating pair during conjugation. These findings offer novel perspectives on gene mobility within the gut microbiota and underscore the potential value of TP114 as an exceptionally effective DNA delivery system for applications related to microbiome genetic engineering [71]. Furthermore, P Ruotsalainen et al. utilized modified and mobilized CRISPR-Cas9-inducing plasmids to alter bacterial communities. Their team designed a midbiotic system composed of a conjugative IncP plasmid RP423 and a mobilized pCas9 plasmid consisting of Streptococcus pyogenes–derived CRISPR/Cas9 that targets conserved sites in two different beta-lactamase genes by plasmid-encoded CRISPR RNA (crRNA). They demonstrated that ESBL-positive transconjugants could effectively lose resistance by conjugative plasmid aligned with a mobilized antibiotic resistance gene targeting CRISPR-plasmid. Thus, various gene types can be targeted together by some CRISPR RNA encoding sections within the transferred plasmids. Their work allowed the inserting or deletion of genes in natural bacteria by CRISPR reality, which could be recognized as a tool for genetic engineering of already existing bacterial communities [72]. In conclusion, these findings introduce conjugative plasmids as a robust and efficient strategy for targeted genetic manipulation of diverse bacterial species from the human gut.

#### Bacteriophage

Phages are bacterial viruses that can infect bacteria. These viruses can be classified into two groups, including lytic or temperate [73]. Their DNA is packed within their capsids and can be injected into host bacteria. The vital aspect of phages is particularly their specificity. Usually, such viruses target some strains in a specific bacterial species and fail to penetrate even the closest ones. This ability to selectively infect bacterial strains is controlled by complex intermolecular interactions among molecules on the cell surface (e.g., proteins, polysaccharides, macromolecular structures, like flagella) and the phage’s host recognition domain (HRD) [74]. BB Hsu et al. discovered a non-invasive strategy for modifying the gene expression of particular bacteria in mammals’ gut microbiomes through oral delivery. First, the temperate phage λ is engineered to express a nuclease-deactivated Cas9 (dCas9), which makes it capable of suppressing gene expression in bacteria both in vitro and in vivo (colonizing the mouse gut). They worked on an aqueous-based capsule with a release mechanism based on microbiota while reducing the possibility of disturbance in host mechanisms, which facilitated the phage with administrative strength. Based on their findings, bacteria’s gene expression within the mammalian gut can be modified by an exact oral dose [75]. D Bikard’s team utilized the RNA-guided nuclease Cas9 delivered by a phage to make programmable, sequence-specific antimicrobials. They show that Cas9, reprogrammed to target virulence genes, kills virulent, but not avirulent, Staphylococcus aureus. To prevent plasmid-borne resistance genes from spreading, reprogramming the nuclease allows it to suppress antibiotic-resistant genes. As a result, staphylococcal plasmids that carry antibiotic-resistance genes will be destroyed without harming non-pathogenic staphylococcal genes. Their investigation revealed that CRISPR-Cas9, characterized by antimicrobial features, can eliminate *S. aureus* in in-vivo conditions, as demonstrated in a colonized mouse skin model. Thus, the phagemid-delivered CRISPR mechanism provides opportunities to modify complex bacterial communities by a sequence-specific procedure. Phagemid delivery is suitable for several applications, but obstacles related to the purity of their content, mass production, and limited host span prevent utilizing them extensively [76]. These findings suggest that phage-based tools could be developed as a powerful tool to precisely modulate gut bacteria and restore a sustainable microbiome in different dysbiosis-associated diseases.

#### Metagenomic alteration of gut microbiome by in situ conjugation (MAGIC)

This procedure differs from CRISPR, directly manipulating gut bacteria in their original habitat through natural horizontal gene transfer to render engineered DNA. Since providing the right conditions for cultivating gut bacteria and applying required changes is difficult in a laboratory environment, MAGIC exploits donor bacteria. Therefore, to conjugate mobile vectors carrying genetically engineered information, it is directly delivered to the gut microbiome. An engineered donor transfers replicative or integrative pGT vectors into prepared recipients within a microbiome composition. Replicative vectors are characterized by a wide range of host’s origin of replication, while integrative vectors possess a transposable Himar cassette and transposase (Tnase). A genetically integrated E. coli strain accounts for one donor carrying genetically integrated conjugative transfer genes (tra) and a mCherry gene (mCh). In addition, Transconjugated bacteria are distinguishable based on the expression of an engineered DNA that contains GFP and an antibiotic-resistance gene (AbR). Necessary improvements of the system include enhancing vector stability so that the donor-strain dosage could authorize finer quantitative and temporal control of preservation of genetic content in situ, which might be productive in short-term or long-term stimulation of engineered functions. Constructing genetic programs regarding recipients’ exact features should be optimized to target the implementation of required tasks in a specific genus existing within a community [77].

#### Environmental Transformation sequencing (ET-Seq)

This mechanism was developed for goal-oriented genome editing of particular organisms in microbial compositions by establishing a new example for microbial modification related to that specific experiment or program for all microbial communities, such as human, environmental, and industrial. ET-Seq function requires a microbial community to be exposed to a randomly integrating mobile genetic matter. When any selection is missing, the whole DNA community can be extracted and sequenced to supply closely related insertion efficiencies for individual microbiome members. Eventually, species-specific genetic availability would be disclosed by percentage measurement of every member of the microbiome community, which attains transposon insertion through bioinformatics and experimental procedures. Furthermore, ET-Seq has been established as a strong tool to analyze and discover horizontal gene transfer in various communities. Finally, DNA-editing All-in-one RNA-guided CRISPR-Cas Transposase (DART) systems were designed and utilized to target the DNA insertion into organisms recognized as tolerable by ET-Seq. This approach provides the ability to both ablate the function of targeted genes and deliver customized genetic cargo in organisms shown to be genetically tractable by ET-Seq. It is capable of assaying the efficacy of CRISPR-Cas-guided transposition into the genome of a target organism by using DART systems that are barcoded and compatible with the same sequencing methods used for ET-Seq. Thus, using DART to target genome editing permits genetic manipulation of distant microbial community members. In conclusion, ET-Seq and DART systems combination lays the foundation of the novel field of in situ microbial genetics [78] (Table 1).

**Table 1:** Major limitations and advantageous of common tools in microbiome genetic engineering

Major tools in microbiome genetic engineering	Major limitations	Major advantages	Ref.
**Conjugative plasmid**	1. Create significant errors or artifacts in the measurement of conjugation rates 2. Dissemination of antibiotic resistance	1. independent operation 2. Generally resistant to bacterial defence mechanisms	[79]
**Bacteriophage**	1. Large libraries of fully characterized phages are necessary for targeting even the most therapeutically important bacterial strains. 2. Development of resistance species 3. mass production 4. purity of their content 5. It is difficult to deliver bacteriophages to the proper location inside a microbiome.	1. possess a limited range of specificity, making them harmless to commensal microbiota 2. Capsids are the outer shells of phages, which guard the DNA or RNA within 3. Extremely stable, usually unaffected by changes in temperature and pH 4. Easy and inexpensive to propagate on bacterial hosts	[80, 81]
**MAGIC**	1. Penetrance of the donor 2. Host variables including effectiveness of genomic integration, plasmid copy number, and etc. might interfere with expression of the target product in recipient cells. 3. Long-term in vivo stability maintenance of genetically designed constructs in complex microbial ecosystems	1. Both Gram- negative and Gram- positive cells are compatible 2. Isolation of genetically modifiable strains from diverse communities	[77, 82]
**ET-Seq**	1. The successful transfer of microbial communities is contingent on the presence of appropriate host organisms to serve as recipients.	1. Is able to evaluate the relative amenability of each bacteria species agnostically within the community to genetic manipulation in a quantitative fashion 2. It is possible to avoid the necessity of culturing and evaluating individual strains within the microbial population.	[83]

### Microbiome genetic engineering in clinical application

The uremic solute indoxyl sulfate has been discovered to be associated with an elevated mortality rate and other h24 armful results, specifically in patients suffering from chronic kidney disease. Furthermore, the tryptophanase gene BT1492 in several *Bacteroides* species, including *B. thetaiotaomicron* (B. theta), produces uremic solutes such as indoxyl sulfate and p-Cresyl sulfate. Researchers colonized germ-free mice with the mutant bacteria Bt Δ1492.5 (carrying a deletion of the relevant tryptophanase gene 1492) in order to investigate if modification of a single bacterial gene can encourage indoxyl sulfate production in vivo. Unlike those with wild-type bacteria, these colonized mice with Bt Δ1492 showed no trace of serum or urinary indoxyl sulfate. Thus, manipulating the manufacture of indoxyl sulfate by genetic modification of the gut microbiota is possible [84]. R D’Souza et al., using plasmid pVE5523 encoding PAMI (peptide blocker: GHWYYRCW), genetically engineered *Lactococcus lactis subsp. lactis IL1403* and *Lactococcus lactis subsp. cremoris MG1363* and fed them to diabetic mice models. They discovered a considerable decline in the blood glucose level by the end of the 20 days trial. Hence, this product can be classified as a biodrug capable of controlling the blood glucose level in diabetic patients [85]. An expression and delivery system founded on recombinant probiotic species *L. paracasei* to act as a live vector for the oral delivery of human ACE2 was developed by A. Verma et al. They demonstrated that codon-optimized human ACE2 could be adequately expressed in *L. paracasei* with enzymatic activity. Another finding of A Verma et al. was that oral administration of recombinant *L. paracasei* expressing the secreted ACE2 in fusion with CTB in mice enhanced serum and tissue ACE2 activities. Another turning point is that oral administration of recombinant ACE2-LP considerably lowers diabetes-induced retinal neurovascular degeneration in two mouse models of DR [86]. CL Ho et al. objective circled reprogrammed commensal *Escherichia coli Nissle 1917* (EcN) to attach to the heparan sulfate proteoglycan (HSPG) on the cancer cell external surface, then secrete myrosinase to alter dietary glucosinolate to sulforaphane that could limit growth and activate apoptosis in cancer cells. First, they enhanced Myrosinase genes for E. coli expression, cloned them into the pET28b expression vector, and transformed them into *E. coli BL21* (DE3). They revealed that genetically engineered microbes and glucosinolates lead to > 95% proliferation inhibition of murine, human, and colorectal adenocarcinoma cell lines in vitro. They show that in mice models of colorectal carcinoma, the combination of a cruciferous vegetable diet and engineered microbes significantly reduced tumor growth and cancer lesion development [87]. Moreover, ML Hanson et al. worked on a method for localized delivery of the immunosuppressive cytokine interleukin (IL)-27 that is actively synthesized in situ by the food-grade bacterium *Lactococcus lactis* (LL-IL-27) and examined its impact on reducing colitis in mice. At first, they synthesized two genes encoding mouse IL-27 with optimum codon use for *L lactis* and joined them by a linker. Then, a signal sequence was introduced to enable product secretion, and the product was added to *L lactis*. From their findings, it can be concluded that genetically engineered *Lactococcus lactis* can be therapeutic in T-cell–dependent chronic enterocolitis, proposing a safer and more effective treatment for IBD patients [88]. E Spisni et al. developed engineered nonpathogenic-invasive *Escherichia coli* (InvColi) strains for anti-COX-2 RNAi (InvColishCOX2). Their objective was to examine the in vivo possibility of a novel COX-2 silencing strategy in a murine model of colitis impelled by dextran sulfate sodium (DSS). Enema administrations of InvColishCOX2 in DSS-treated mice resulted in various outcomes such as COX-2 downregulation, colonic mucosa conservation, decreased colitis disease activity index (DAI), and an increase in the number of survived mice. Furthermore, DSS/InvColishCOX2-treated mice showed fewer signs of circulating pro-inflammatory cytokines and a lessened colitis-assisted switch of gut microbiota. Thus, the InvColishCOX2 strategy could be rewarding for molecularly treating intestinal inflammatory illnesses [89].

#### Microbiome engineering via Extracellular vesicle

**Fig. 4:**
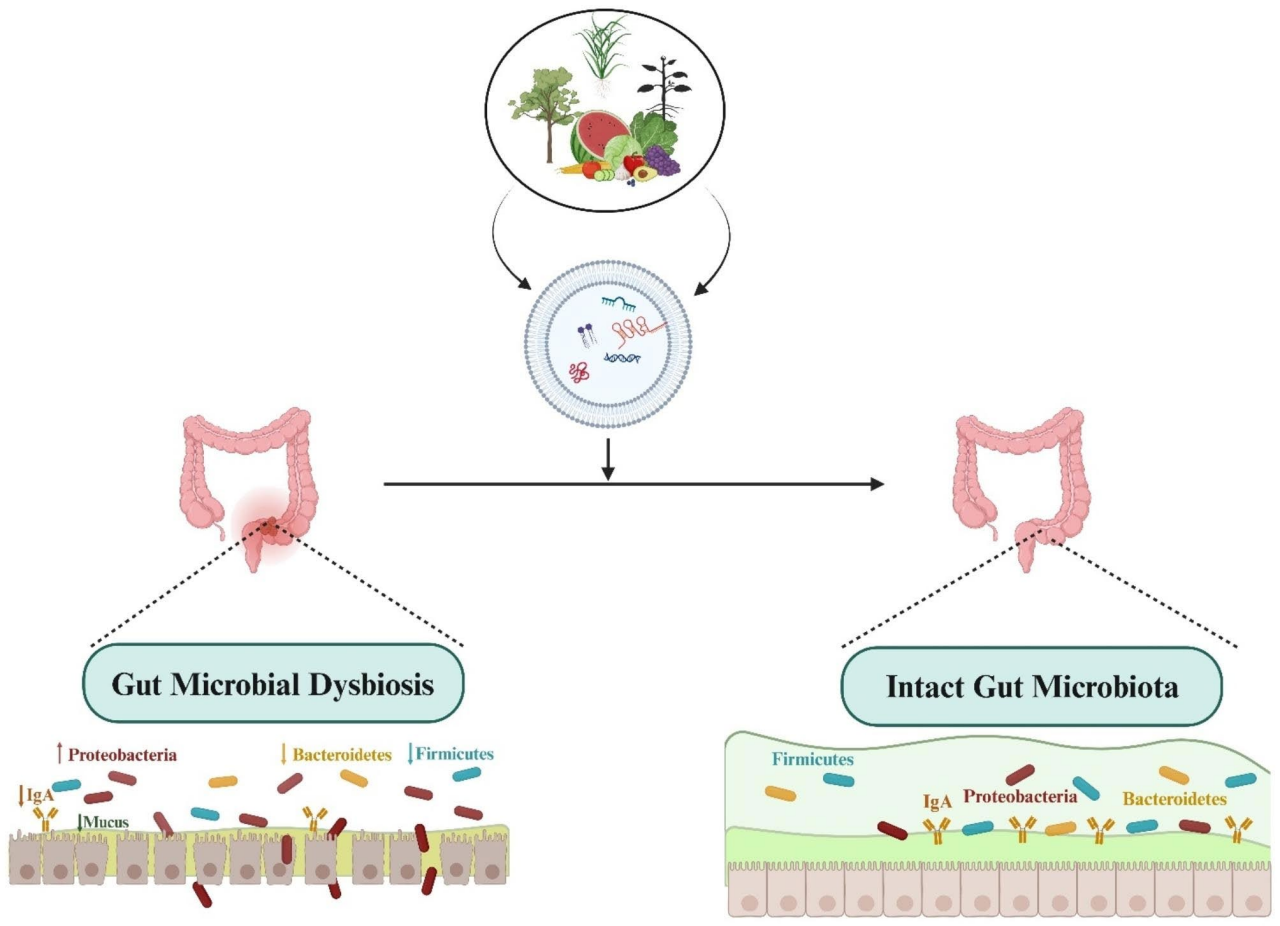
A schematic representation of plant-derived extracellular vesicle in microbiome engineering

Plant exosomes are membranous structures originating from eukaryotic plant cells. These structures have a size ranging from 30 to 150 nanometers and consist of multiple vesicles. Plant exosomes refer to vesicles with a two-layered membranous structure made up of lipids. These vesicles contain biologically active proteins, lipids, and RNA. They play a crucial role in transmitting intercellular information and have been found to regulate several physiological processes, such as intestinal diseases, cancer, and the immune system. In vivo, exosome-like nanovesicles (ELNs) derived from plants present a reduced immune risk and do not induce an elevation in the production of pro-inflammatory cytokines [90]. According to a recent inquiry, plant vesicles may be able to modify the gastrointestinal tract’s microbial composition, thereby indicating their potential utility in addressing intestinal dysbiosis and associated disorders. In this regard, Zhang et al. investigated whether *Taraxacum officinale* (*T. officinale*)-derived ELNs exerted hypotensive effects in intermittent hypoxia (IH)-induced hypertensive disorders and their potential mechanisms. Initially, the researchers produced natural nanoparticles sourced from *T. officinale* that possessed desirable dimensions and exhibited a consistently negative surface charge. These particles were rich in lipids and also contained several functional proteins. Their study observed that ELNs significantly reduced hypertension caused by IH and displayed remarkable anti-inflammatory properties on intestinal tissues in rats with IH-induced hypertension. The administration of ELNs also decreased in intestinal tissue damage, specifically the loss of goblet cells and compromised barrier integrity, ultimately inhibiting the systemic inflammatory response. In addition, the researchers analyzed the intestinal microbial composition and the content of SCFAs, wherein they discovered significant alterations in the structure and diversity of the intestinal microbial communities. Notably, the key factor associated with the observed differences in the flora was identified as butyrate. In this manner, using *T. officinale*-derived ELNs was efficacious in mitigating hypertension caused by IH. Mechanistically, the beneficial effects of ELNs were mediated through modulation of the microbiome and the resultant increase in butyrate levels [91]. As well, Teng et al. developed ginger exosome-like nanoparticles (GELNs) that delivered their microRNAs to Lactobacillus rhamnosus (LGG), altering the bacteria’s gene expression. Lactobacillus rhamnosus preferred absorption behavior was lipid-dependent, and GELN-derived lipids were enriched with 1, 2-dilinoleoyl-sn-glycero-3- phosphate, C18:1/C18:3 (36:4), and 1-palmitoyl-2-linoleoyl-sn-glycero-3-phosphate, C16:0/C18:2 (34:2). GELN-derived mdo-miR7267-3p has a potential binding site for mRNA encoding LGG monooxygenase ycnE. Accordingly, GELN-RNAs are capable of inhibiting ycnE gene expression, elevating indole-3- carboxaldehyde (an identified aryl hydrocarbon receptor ligand), and promoting I3A and IL-22 production, thereby ameliorating colitis [92](Fig. 4).

## Conclusion and future perspectives

The gut microbiota community consists of many living organisms, such as bacteria, archaea, fungi, and small arthropods. These organisms form and interact together and could influence the host’s health. Recent findings utilizing molecular techniques consisting of sequencing of 16 S rRNA genes have distinguished alterations in the composition and action of the gut microbiome accompanied by various diseases. Developing advanced strategies to manipulate the gut microbiome one by one or as a community to enhance human health is immense. Genome editing using CRISPR-Cas9 opens up new ways to modify the gut microbiome and understand its function of specific components. The genetic engineering of microbiomes has recently become an area of interest for researchers since it provides solutions to a significant health problem. In this context, recent experimentation demonstrated that strategies including conjugative plasmids, bacteriophage, MAGIC, and ET-Seq could be effective in the genetic modification of the microbiome. However, there have been obstacles in microbiome genetic engineering. Due to their low abundance in the intestinal tract (e.g., *E. coli* or *L. lactis*) and inability to effectively colonize the gut, these modified bacteria cannot continually supply the required therapeutic compound at large concentrations (e.g., EcN). Therefore, these bacteria’s activity is frequently restricted to temporary effects: as they are cleared from the body, their therapeutic benefits may fade. Another critical difficulty is safety. In the long run, the functional integrity of host immunity will decay due to their microbiome overdependence as a means of defense against pathogens, toxins, and cancers. Furthermore, spontaneous mutations are unavoidable characteristics of each live cell. It frequently results in carcinogenesis, microbial drug resistance, and virulent prototype exaltation. Hence, beneficial microbes may mutate and turn up as pathogenic or carcinogenic.

Nanoparticles (NPs) are among the structurally and dynamically altered nano-medicines interacting with their environment [93]. They usually use chemical bonds and reactions to improve their structure at their designated drug release site. Targeted methods of delivery are becoming more abundant due to technological advancements. Production of nanoparticles could use a short-step synthesis process, usually using polyethylene glycol (PEG) and polycaprolactone (PCL), polymers of FDA-approved products. These molecules possess a high capacity for drug loading. The interior fold may be filled with minimal influence on drug delivery or molecular behavior, allowing for an effective and safe delivery system to be constructed. Their lengthy polymeric chains can shield the payload from immune cells or antibodies before it reaches its activation site. Engineered nanoparticles or biomaterials have demonstrated the ability to modulate the gut microbiota for treating inflammatory diseases such as colorectal cancer. In this context, Lee et al describe the creation of a nanomedicine comprised of hyaluronic acid and bilirubin (HABN). This novel compound selectively accumulates in inflamed colonic epithelial cells, effectively repairing the damaged epithelial barrier in a murine model with acute colitis. Surprisingly, HABN can regulate the composition of the gut microbiota by increasing its overall richness and diversity. Moreover, it significantly enhances *Akkermansia muciniphila* and *Clostridium* XIVα abundance, two microorganisms that are vital in maintaining gut homeostasis. Importantly, HABN was found to be linked with pro-inflammatory macrophages and was able to regulate innate immune responses, ultimately demonstrating significant therapeutic efficacy in treating colitis. Therefore, their findings shed valuable light on the influence of nanotherapeutics on gut homeostasis, microbiome engineering, and innate immune responses in the context of treating inflammatory diseases [94]. Thereby, NPs show great promise for engineering of microbiota or even potentially live biotherapeutic products as treatment for inflammatory diseases such as cancer and diabetes.

## Data Availability

The datasets used and/or analyzed during the current study are available from the corresponding author on reasonable request.

## References

[1] Zhao N, Liu Z, Xing J, Zheng Z, Song F, Liu S (2022). A novel strategy for high-specificity, high-sensitivity, and high-throughput study for gut microbiome metabolism of aromatic carboxylic acids. Chin Chem Lett.

[2] Gilbert JA, Blaser MJ, Caporaso JG, Jansson JK, Lynch SV, Knight R (2018). Current understanding of the human microbiome. Nat Med.

[3] Zhang F, Lau RI, Liu Q, Su Q, Chan FK, Ng SC (2023). Gut microbiota in COVID-19: key microbial changes, potential mechanisms and clinical applications. Nat Reviews Gastroenterol Hepatol.

[4] Calder PC, Ortega EF, Meydani SN, Adkins Y, Stephensen CB, Thompson B (2022). Nutrition, immunosenescence, and infectious disease: an overview of the scientific evidence on micronutrients and on modulation of the gut microbiota. Adv Nutr.

[5] Ma X, Zhou Z, Zhang X, Fan M, Hong Y, Feng Y (2020). Sodium butyrate modulates gut microbiota and immune response in colorectal cancer liver metastatic mice. Cell Biol Toxicol.

[6] Jagielski P, Łuszczki E, Wnęk D, Micek A, Bolesławska I, Piórecka B (2022). Associations of nutritional behavior and gut microbiota with the risk of COVID-19 in healthy young adults in Poland. Nutrients.

[7] Amiri P, Hosseini SA, Ghaffari S, Tutunchi H, Ghaffari S, Mosharkesh E (2022). Role of butyrate, a gut microbiota derived metabolite, in cardiovascular diseases: a comprehensive narrative review. Front Pharmacol.

[8] Quaglio AEV, Grillo TG, De Oliveira ECS, Di Stasi LC, Sassaki LY (2022). Gut microbiota, inflammatory bowel disease and colorectal cancer. World J Gastroenterol.

[9] Walker AW, Hoyles L (2023). Human microbiome myths and misconceptions. Nat Microbiol.

[10] Marsh JW, Kirk C, Ley RE. Toward Microbiome Engineering: expanding the repertoire of genetically tractable members of the human gut Microbiome. Annu Rev Microbiol. 2023;77. 10.1146/annurev-micro-032421-112304 37339736

[11] Rutter JW, Dekker L, Owen KA, Barnes CP (2022). Microbiome engineering: Engineered live biotherapeutic products for treating human disease. Front Bioeng Biotechnol.

[12] Hu B, Das P, Lv X, Shi M, Aa J, Wang K (2022). Effects of ‘Healthy’Fecal microbiota transplantation against the deterioration of Depression in Fawn-Hooded rats. Msystems.

[13] Pan Z-Y, Zhong H-J, Huang D-N, Wu L-H, He X-X. Beneficial effects of repeated washed microbiota transplantation in children with autism. Front Pead. 2022:971. 10.3389/fped.2022.928785 PMC9249087 35783298

[14] Rinninella E, Raoul P, Cintoni M, Franceschi F, Miggiano GAD, Gasbarrini A (2019). What is the healthy gut microbiota composition? A changing ecosystem across age, environment, diet, and diseases. Microorganisms.

[15] Koliada A, Moseiko V, Romanenko M, Piven L, Lushchak O, Kryzhanovska N (2020). Seasonal variation in gut microbiota composition: cross-sectional evidence from ukrainian population. BMC Microbiol.

[16] Davenport ER, Mizrahi-Man O, Michelini K, Barreiro LB, Ober C, Gilad Y (2014). Seasonal variation in human gut microbiome composition. PLoS ONE.

[17] Manor O, Dai CL, Kornilov SA, Smith B, Price ND, Lovejoy JC (2020). Health and disease markers correlate with gut microbiome composition across thousands of people. Nat Commun.

[18] Johnson KV-A (2020). Gut microbiome composition and diversity are related to human personality traits. Hum Microbiome J.

[19] Dąbrowska K, Witkiewicz W (2016). Correlations of host genetics and gut microbiome composition. Front Microbiol.

[20] Al Bander Z, Nitert MD, Mousa A, Naderpoor N (2020). The gut microbiota and inflammation: an overview. Int J Environ Res Public Health.

[21] Russo E, Giudici F, Ricci F, Scaringi S, Nannini G, Ficari F (2021). Diving into inflammation: a pilot study exploring the dynamics of the immune–microbiota axis in ileal tissue layers of patients with Crohn’s disease. J Crohn’s Colitis.

[22] Sanders DJ, Inniss S, Sebepos-Rogers G, Rahman FZ, Smith AM. The role of the microbiome in gastrointestinal inflammation. Biosci Rep. 2021;41(6). 10.1042/BSR20203850 PMC8201460 34076695

[23] Perez-Lopez A, Behnsen J, Nuccio S-P, Raffatellu M (2016). Mucosal immunity to pathogenic intestinal bacteria. Nat Rev Immunol.

[24] Hiemstra IH. Microbial control of the mucosal barrier function. 2013.

[25] Honda K, Littman DR (2012). The microbiome in infectious disease and inflammation. Annu Rev Immunol.

[26] Liu J, Liu Z, Pang Y, Zhou H (2022). The interaction between nanoparticles and immune system: application in the treatment of inflammatory diseases. J Nanobiotechnol.

[27] Gill PA, Inniss S, Kumagai T, Rahman FZ, Smith AM (2022). The role of diet and gut microbiota in regulating gastrointestinal and inflammatory disease. Front Immunol.

[28] Dehghan M, Ghorbani F, Najafi S, Ravaei N, Karimian M, Kalhor K et al. Progress toward Molecular Therapy for Diabetes Mellitus: A Focus on Targeting Inflammatory Factors. Diabetes Research and Clinical Practice. 2022:109945. 10.1016/j.diabres.2022.109945 35690269

[29] Zhang X, Qu Y-Y, Liu L, Qiao Y-N, Geng H-R, Lin Y (2021). Homocysteine inhibits pro-insulin receptor cleavage and causes insulin resistance via protein cysteine-homocysteinylation. Cell Rep.

[30] Hoorzad P, Mousavinasab F, Tofigh P, Kalahroud EM, Aghaei-Zarch SM, Salehi A et al. Understanding the lncRNA/miRNA-NFκB regulatory network in diabetes Mellitus: from function to clinical translation. Diabetes Res Clin Pract. 2023:110804. 10.1016/j.diabres.2023.110804 37369279

[31] Zeinali F, Aghaei Zarch SM, Jahan-Mihan A, Kalantar SM, Vahidi Mehrjardi MY, Fallahzadeh H (2021). Circulating microRNA-122, microRNA-126-3p and microRNA-146a are associated with inflammation in patients with pre-diabetes and type 2 diabetes mellitus: a case control study. PLoS ONE.

[32] Dehghani M, Zarch SMA, Mehrjardi MYV, Nazari M, Babakhanzadeh E, Ghadimi H (2020). Evaluation of miR-181b and mir-126-5p expression levels in T2DM patients compared to healthy individuals: relationship with NF-κB gene expression. Endocrinología. Diabetes y Nutrición.

[33] Zarch SMA, Tezerjani MD, Talebi M, Mehrjardi MYV (2020). Molecular biomarkers in diabetes mellitus (DM). Med J Islamic Repub Iran.

[34] Zeinali F, Aghaei Zarch SM, Vahidi Mehrjardi MY, Kalantar SM, Jahan-Mihan A, Karimi-Nazari E (2020). Effects of synbiotic supplementation on gut microbiome, serum level of TNF-α, and expression of microRNA-126 and microRNA-146a in patients with type 2 diabetes mellitus: study protocol for a double-blind controlled randomized clinical trial. Trials.

[35] Mirzavandi F, Babaie S, Rahimpour S, Razmpoosh E, Talenezhad N, Zarch SMA (2020). The effect of high dose of intramuscular vitamin D supplement injections on depression in patients with type 2 diabetes and vitamin D deficiency: a randomized controlled clinical trial. Obes Med.

[36] Aghaei M, Khodadadian A, Elham K-N, Nazari M, Babakhanzadeh E (2020). Major miRNA involved in insulin secretion and production in beta-cells. Int J Gen Med.

[37] Das T, Jayasudha R, Chakravarthy S, Prashanthi GS, Bhargava A, Tyagi M (2021). Alterations in the gut bacterial microbiome in people with type 2 diabetes mellitus and diabetic retinopathy. Sci Rep.

[38] Pordel S, Khorrami M, Saadatpour F, Rezaee D, Cho WC, Jahani S et al. The role of microRNA-185 in the Pathogenesis of Human Diseases: a Focus on Cancer. Pathology-Research and Practice. 2023:154729. 10.1016/j.prp.2023.154729 37639952

[39] Faramin Lashkarian M, Hashemipour N, Niaraki N, Soghala S, Moradi A, Sarhangi S (2023). MicroRNA-122 in human cancers: from mechanistic to clinical perspectives. Cancer Cell Int.

[40] Fattahi M, Shahrabi S, Saadatpour F, Rezaee D, Beyglu Z, Delavari S et al. microRNA-382 as a tumor suppressor? Roles in tumorigenesis and clinical significance. Int J Biol Macromol. 2023:125863. 10.1016/j.ijbiomac.2023.125863 37467828

[41] Gao Y, Zhang H, Lirussi F, Garrido C, Ye X-Y, Xie T (2020). Dual inhibitors of histone deacetylases and other cancer-related targets: a pharmacological perspective. Biochem Pharmacol.

[42] Sui X, Zhang R, Liu S, Duan T, Zhai L, Zhang M (2018). RSL3 drives ferroptosis through GPX4 inactivation and ROS production in colorectal cancer. Front Pharmacol.

[43] Zhao H, Ming T, Tang S, Ren S, Yang H, Liu M (2022). Wnt signaling in colorectal cancer: pathogenic role and therapeutic target. Mol Cancer.

[44] Najafi S, Khatami SH, Khorsand M, Jamali Z, Shabaninejad Z, Moazamfard M et al. Long non-coding RNAs (lncRNAs); roles in tumorigenesis and potentials as biomarkers in cancer diagnosis. Exp Cell Res. 2022:113294. 10.1016/j.yexcr.2022.113294 35870535

[45] Shirvani H, Ghanavi J, Aliabadi A, Mousavinasab F, Talebi M, Majidpoor J (2023). MiR-211 plays a dual role in cancer development: from tumor suppressor to tumor enhancer. Cell Signal.

[46] Khasraghi LB, Nouri M, Vazirzadeh M, Hashemipour N, Talebi M, Zarch FA (2023). MicroRNA-206 in human cancer: mechanistic and clinical perspectives. Cell Signal.

[47] Lu L, Dong J, Liu Y, Qian Y, Zhang G, Zhou W et al. New insights into natural products that target the gut microbiota: Effects on the prevention and treatment of colorectal cancer. Front Pharmacol. 2022;13. 10.3389/fphar.2022.964793 PMC9420899 36046819

[48] Haque S, Raina R, Afroze N, Hussain A, Alsulimani A, Singh V, et al. editors. Microbial dysbiosis and epigenetics modulation in cancer development–A chemopreventive approach. Seminars in Cancer Biology; 2021: Elsevier. 10.1016/j.semcancer.2021.06.024 34216789

[49] Ferreira RM, Pereira-Marques J, Pinto-Ribeiro I, Costa JL, Carneiro F, Machado JC (2018). Gastric microbial community profiling reveals a dysbiotic cancer-associated microbiota. Gut.

[50] Zhang J, Zhang F, Zhao C, Xu Q, Liang C, Yang Y (2019). Dysbiosis of the gut microbiome is associated with thyroid cancer and thyroid nodules and correlated with clinical index of thyroid function. Endocrine.

[51] Niccolai E, Russo E, Baldi S, Ricci F, Nannini G, Pedone M (2021). Significant and conflicting correlation of IL-9 with Prevotella and Bacteroides in human colorectal cancer. Front Immunol.

[52] Zou S, Fang L, Lee M-H (2018). Dysbiosis of gut microbiota in promoting the development of colorectal cancer. Gastroenterol Rep.

[53] Lu C-c, Hu Z-b, Wang R, Hong Z-h, Lu J, Chen P-p (2020). Gut microbiota dysbiosis-induced activation of the intrarenal renin–angiotensin system is involved in kidney injuries in rat diabetic nephropathy. Acta Pharmacol Sin.

[54] Xu H, Zhao H, Fan D, Liu M, Cao J, Xia Y et al. Interactions between gut microbiota and immunomodulatory cells in rheumatoid arthritis. Mediators of Inflammation. 2020;2020. 10.1155/2020/1430605 PMC7499318 32963490

[55] Fardi F, Khasraghi LB, Shahbakhti N, Naseriyan AS, Najafi S, Sanaaee S et al. An interplay between non-coding RNAs and gut microbiota in human health. Diabetes Res Clin Pract. 2023:110739. 10.1016/j.diabres.2023.110739 37270071

[56] Albright MB, Louca S, Winkler DE, Feeser KL, Haig S-J, Whiteson KL (2022). Solutions in microbiome engineering: prioritizing barriers to organism establishment. ISME J.

[57] Ji B, Nielsen J (2015). From next-generation sequencing to systematic modeling of the gut microbiome. Front Genet.

[58] D’Haens GR, Jobin C (2019). Fecal microbial transplantation for diseases beyond recurrent clostridium difficile infection. Gastroenterology.

[59] Cunningham M, Azcarate-Peril MA, Barnard A, Benoit V, Grimaldi R, Guyonnet D (2021). Shaping the future of probiotics and prebiotics. Trends Microbiol.

[60] Inda ME, Broset E, Lu TK, de la Fuente-Nunez C (2019). Emerging frontiers in microbiome engineering. Trends Immunol.

[61] Angelakis E (2017). Weight gain by gut microbiota manipulation in productive animals. Microb Pathog.

[62] Lawson CE (2021). Retooling microbiome engineering for a sustainable future. Msystems.

[63] Foo JL, Ling H, Lee YS, Chang MW (2017). Microbiome engineering: current applications and its future. Biotechnol J.

[64] Najafi S, Zarch SMA, Majidpoor J, Pordel S, Aghamiri S, Rasul MF et al. Recent insights into the roles of circular RNAs in human brain development and neurologic diseases. Int J Biol Macromol. 2022. 10.1016/j.ijbiomac.2022.11.166 36410538

[65] Chávez-Granados PA, Manisekaran R, Acosta-Torres LS, Garcia-Contreras R. CRISPR/Cas gene-editing technology and its advances in dentistry. Biochimie. 2021. 10.1016/j.biochi.2021.12.012 34974144

[66] Zhou X, Wang X, Luo H, Wang Y, Wang Y, Tu T (2021). Exploiting heterologous and endogenous CRISPR-Cas systems for genome editing in the probiotic Clostridium butyricum. Biotechnol Bioeng.

[67] Zheng L, Tan Y, Hu Y, Shen J, Qu Z, Chen X, et al. CRISPR/Cas-Based genome editing for human gut commensal Bacteroides species. ACS Synthetic Biology; 2022. 10.1021/acssynbio.1c00543 34990118

[68] Shin J, Kang S, Song Y, Jin S, Lee JS, Lee J-K (2019). Genome engineering of Eubacterium limosum using expanded genetic tools and the CRISPR-Cas9 system. ACS Synth Biol.

[69] Armianinova D, Karpov D, Kotliarova M, Goncharenko A (2022). Genetic Engineering in Mycobacteria. Mol Biol.

[70] Gambetta GA, Lagarias JC. Genetic engineering of phytochrome biosynthesis in bacteria. Proceedings of the National Academy of Sciences. 2001;98(19):10566-71. 10.1073/pnas.191375198 PMC58506 11553807

[71] Neil K, Allard N, Grenier F, Burrus V, Rodrigue S (2020). Highly efficient gene transfer in the mouse gut microbiota is enabled by the Incl2 conjugative plasmid TP114. Commun Biology.

[72] Ruotsalainen P, Penttinen R, Mattila S, Jalasvuori M (2019). Midbiotics: conjugative plasmids for genetic engineering of natural gut flora. Gut Microbes.

[73] Chibani CM, Farr A, Klama S, Dietrich S, Liesegang H (2019). Classifying the unclassified: a phage classification method. Viruses.

[74] Gibb B, Hyman P, Schneider CL (2021). The many applications of engineered bacteriophages—An overview. Pharmaceuticals.

[75] Hsu BB, Plant IN, Lyon L, Anastassacos FM, Way JC, Silver PA (2020). In situ reprogramming of gut bacteria by oral delivery. Nat Commun.

[76] Bikard D, Euler CW, Jiang W, Nussenzweig PM, Goldberg GW, Duportet X (2014). Exploiting CRISPR-Cas nucleases to produce sequence-specific antimicrobials. Nat Biotechnol.

[77] Ronda C, Chen SP, Cabral V, Yaung SJ, Wang HH (2019). Metagenomic engineering of the mammalian gut microbiome in situ. Nat Methods.

[78] Rubin BE, Diamond S, Cress BF, Crits-Christoph A, He C, Xu M et al. Targeted genome editing of bacteria within microbial communities. bioRxiv. 2020.

[79] Wang X, Zhang H, Long X, Xu X, Ren H, Mao D (2023). Global increase of Antibiotic Resistance genes in conjugative plasmids. Microbiol Spectr.

[80] Łobocka M, Dąbrowska K, Górski A (2021). Engineered bacteriophage therapeutics: rationale, challenges and future. BioDrugs.

[81] Voorhees PJ, Cruz-Teran C, Edelstein J, Lai SK (2020). Challenges & opportunities for phage-based in situ microbiome engineering in the gut. J Controlled Release.

[82] Whitfill T, Oh J (2019). Recoding the metagenome: microbiome engineering in situ. Curr Opin Microbiol.

[83] Mozumdar D, Csörgő B, Bondy-Denomy J (2022). Genetic manipulation of a CAST of characters in a Microbial Community. CRISPR J.

[84] Yacoub R, Wyatt CM (2017). Manipulating the gut microbiome to decrease uremic toxins. Kidney Int.

[85] D’Souza R, Pandeya DR, Rahman M, Lee HS, Jung J-K, Hong S-T (2012). Genetic engineering of Lactococcus lactis to produce an amylase inhibitor for development of an anti-diabetes biodrug. New Microbiol.

[86] Verma A, Xu K, Du T, Zhu P, Liang Z, Liao S (2019). Expression of human ACE2 in Lactobacillus and beneficial effects in diabetic retinopathy in mice. Mol Therapy-Methods Clin Dev.

[87] Ho CL, Tan HQ, Chua KJ, Kang A, Lim KH, Ling KL (2018). Engineered commensal microbes for diet-mediated colorectal-cancer chemoprevention. Nat Biomedical Eng.

[88] Hanson ML, Hixon JA, Li W, Felber BK, Anver MR, Stewart CA (2014). Oral delivery of IL-27 recombinant bacteria attenuates immune colitis in mice. Gastroenterology.

[89] Spisni E, Valerii MC, De Fazio L, Cavazza E, Borsetti F, Sgromo A (2015). Cyclooxygenase-2 silencing for the treatment of colitis: a combined in vivo strategy based on RNA interference and engineered Escherichia coli. Mol Ther.

[90] Nemati M, Singh B, Mir RA, Nemati M, Babaei A, Ahmadi M (2022). Plant-derived extracellular vesicles: a novel nanomedicine approach with advantages and challenges. Cell Communication and Signaling.

[91] Zhang X, Pan Z, Wang Y, Liu P, Hu K (2023). Taraxacum officinale-derived exosome-like nanovesicles modulate gut metabolites to prevent intermittent hypoxia-induced hypertension. Biomed Pharmacother.

[92] Teng Y, Ren Y, Sayed M, Hu X, Lei C, Kumar A (2018). Plant-derived exosomal microRNAs shape the gut microbiota. Cell Host Microbe.

[93] Shirvani H, Jafari H, Moravveji SS, Faranghizadeh FA, Talebi M, Ghanavi J et al. Non-coding RNA in SARS-CoV-2: progress toward therapeutic significance. Int J Biol Macromol. 2022. 10.1016/j.ijbiomac.2022.09.105 PMC9492401 36152703

[94] Lee Y, Sugihara K, Gillilland MG, Jon S, Kamada N, Moon JJ (2020). Hyaluronic acid–bilirubin nanomedicine for targeted modulation of dysregulated intestinal barrier, microbiome and immune responses in colitis. Nat Mater.

